# Therapeutic effects of *Balanites aegyptiaca* DEL extract on diabetes mellitus: a systematic review

**DOI:** 10.3389/fcdhc.2025.1651789

**Published:** 2025-09-02

**Authors:** Olubukola Adebisi Odeniran, Adegboyega Moses Oyefabi, Ifeoluwa Temitayo Oyeyemi, Adewale Adegboyega Oke, Grace Aziken, Grace Adebayo-Gege, Peter Ifeoluwa Adegbola, Lawrence Dayo Adedayo, Olunike Rebecca Abodunrin, Folahanmi Tomiwa Akinsolu, Olajide Odunayo Sobande

**Affiliations:** ^1^ Department of Medicinal Chemistry & amp, Quality Control, National Institute for Pharmaceutical Research and Development (NIPRD), Abuja, Nigeria; ^2^ Department of Community Medicine, Kaduna State University, Kaduna, Nigeria; ^3^ Department of Biosciences and Biotechnology, University of Medical Sciences, Ondo, Nigeria; ^4^ Department of Medical Laboratory Science, McPherson University, Seriki Sotayo, Ogun State, Nigeria; ^5^ Department of Computer Science, University of Benin, Benin, Nigeria; ^6^ Department of Physiology, Faculty of Basic Medical Sciences Baze University, Abuja, Nigeria; ^7^ Department of Biochemistry and Forensic Science, Faculty of Natural and Applied Sciences, Abiola Ajimobi Technical University, Ibadan, Nigeria; ^8^ Department of Human Physiology, Faculty of Basic Medical Sciences, College of Health Sciences, Federal University Wukari, Wukari, Taraba, Nigeria; ^9^ Department of Epidemiology and Health Statistics, Nanjing Medical University, Nanjing, China; ^10^ Center for Reproduction and Population Health Studies, Nigerian Institute of Medical Research, Lagos, Nigeria; ^11^ Clinical Sciences Department, Nigerian Institute of Medical Research, Lagos, Nigeria; ^12^ Nigerian Institute of Medical Research Foundation, Lagos, Nigeria

**Keywords:** Balanites aegyptiaca, diabetes mellitus, therapeutics effects, systematic review, animal experiment

## Abstract

**Background:**

Diabetes mellitus (DM) is a major cause of morbidity and mortality globally as it is associated with long-term health complications which affect the quality of life. Several plants are used in traditional medicine to manage diabetes, with claims of efficacy from traditional healers. One such plant is *Balanites aegyptiaca* (L.) Delile commonly called Desert Date. This systematic review examines the therapeutic effect of *B.aegyptiaca* on diabetes mellitus.

**Methods:**

The protocol for the systematic review was registered with PROSPERO (CRD42024587444). Four databases were searched for articles from 1986 to 1^st^ August 2024. Keywords related to “therapeutic effect”, “*Balanites aegyptiaca*” and “diabetes mellitus” were used. Studies included were all animal models. Each article was critically appraised by two independent reviewers for their methodological quality using the Joanna Briggs Institute Case Control Checklist. The Cochrane SYRICLE Risk of bias tool was used for risk of bias assessment in these animal intervention studies. The animal experiments were conducted mainly in Alloxan- and streptozotocin-induced rat/mice diabetes and a control of non-diabetes induced rats.

**Result:**

A total of 32 articles were included. All the studies were appraised for blood glucose levels, and a reduction in blood glucose was reported in all *in vivo* studies, regardless of the plant part used. Significant decrease in blood glucose level was recorded in Alloxan- and streptozotocin-induced rat/mice diabetes. All the studies reported reduced blood glucose, reduced levels of lipids, reduced weight and increased insulin production. *B. aegyptiaca* mitigated hyperglycaemia irrespective of the presentation form, which includes extract and meal supplementation in rodents, oral capsule intake, and tea or fruit consumption in humans. Various mechanisms, including modulation of glucose metabolizing enzymes, were reported to underlie the *B. aegyptiaca* antidiabetic effect.

**Conclusions:**

Repeated administration of different parts of *B. aegyptiaca* in different presentation forms controlled hyperglycaemia in animal-models. A full-phase clinical trial is needed to determine the therapeutic effects of *B. aegyptiaca* in humans.

**Systematic review registration:**

https://www.crd.york.ac.uk/prospero/, identifier CRD42024587444.

## 1 Introduction

Diabetes mellitus (DM) is a metabolic disorder resulting either from the lack of insulin, impaired insulin action or both, eventually resulting in hyperglycemia (1). Globally, one in every 10 adults has diabetes (2). DM can be classified into type 1, type 2, gestational, hybrid and other specific types (3), with type 2 accounting for more than 90% of DM (4). It is a chronic debilitating disease associated with poor quality of life and high mortality (2, 5, 6).

The management of DM includes lifestyle modification such as exercise, dietary modification and drug therapy (7). Oral synthetic hypoglycemic drugs are effective in controlling hyperglycaemia but are associated with adverse effects such as hepatic toxicity, weight gain, abdomen enlargement and gastrointestinal discomfort (8–10). Alternative therapies, including medicinal plants, have proven efficacy with minimal side effects (11).

Several plants, especially in developing countries have been used by traditional healers for the management of different ailments. One of such plants; *Balanites aegyptiaca* DEL, commonly called Desert date has been used to manage patients with diabetes mellitus (12, 13). It is a deep-rooted, evergreen, semi-deciduous tree up to 12 meters high, widely distributed in Africa and across the Sahel Savannah (12, 13). It is used traditionally to manage several diseases and has been reported to exhibit several pharmacological activities, including antidiabetes, anticancer, anti-inflammatory, antioxidant, antiangiogenic and antimicrobial effects (14, 15). There have been many studies on the antidiabetic effects of the different parts of *B. aegyptiaca* (16–40). The aim of this systematic review is to critically appraise the available evidence on the efficacy of *Balanites aegyptiaca* for managing type 2 DM.

## 2 Methods

### 2.1 Protocol registration

The proposed study was registered as a systematic review with the Prospero Protocol Registration ID Number CRD42024587444 and reported according to PRISMA 2020 checklist (41), pg. 4-5]. (**Supplementary 1**).

### 2.2 Research questions

1. What proportions of previous studies reported hypoglycemic effects when *Balanites aegyptiaca DEL* were used in experimental animals to induce type 2 diabetes mellitus?

2. What were the other medicinal effects when Balanites aegyptiaca DEL were used in experimental animals?

### 2.3 Main study outcome

#### 2.3.1 Primary outcome

Level of blood glucose post administration of the *Balanites aegyptiaca* DEL on experimental animals.

#### 2.3.2 Secondary outcome

This includes other ancillary outcomes such blood insulin level, lipid profile and liver function test post-administration of the *Balanites aegyptiaca* DEL on experimental animals.

### 2.4 Search strategy

We used the population, intervention, comparison and outcome (PICO) framework to guide the search for relevant article for this systematic review (**Table 1**). Three databases (Web of Science, Scopus, PubMed) and Google Scholar were searched for articles from 1986 to 1^st^ August 2024. The PubMed search strategy is presented in the Supplementary materials (**Supplementary File 2**). We used medical subject headings (MeSH) keywords and free text for the PubMed search. The two were combined with Boolean operators “AND” and “Or”. Keywords related to “therapeutic effect”, “*Balanites aegyptiaca*”, and “diabetes mellitus” were also used. Search terms include “Therapeutic effect” OR “Medicinal effect” OR “Benefits” OR “Biological activity” OR “Side effect” OR “Therapeutic biological activity” OR “Antidiabetic”, AND “*Balanites aegyptiaca*” OR “Desert date” AND “Diabetes mellitus” OR “Type 2 diabetes” OR “Type 1 diabetes” OR “Non-insulin-dependent diabetes” OR “Diabet*”.

**Table 1:** Pico Framework utilized for the systematic review.

PICO FRAMEWORK	Description
Population	All animals induced with type 2 diabetes mellitus during the study
Intervention	The animal experiments were conducted mainly in Alloxan- and streptozotocin-induced rat/mice diabetes
Comparison	Controls; which included rats not induced with diabetes mellitus
Outcome	Proportion of the experiment rats with hypoglycemic effects Proportion of experimental rats with change in Liver functions Proportion of experimental rats with reduced weight, increased insulin or any other systemic effect

### 2.5 Inclusion criteria

All animal studies that reported therapeutic effects of *Balanites aegyptiaca* DEL with experimental evidence carried out using any parts of *Balanites aegyptiaca* DEL with hypoglycemic indices such as reduction in plasma glucose level or hypoglycaemia were included. Research on cells lines or human subjects were not included in this systematic review.

### 2.6 Exclusion criteria

Studies were excluded if they were abstracts only or review articles. The titles and abstracts were used to screen the articles. The full texts that lacked primary data, well-defined methodology, duplication, missing information, or similar studies were excluded from the study. The exclusion criteria included review studies, studies that combined *B. aegyptiaca* with other plants, studies that examined the effects of *Balanites aegyptiaca* on conditions other than diabetes mellitus, studies with incomplete or missing information and duplicate publications.

### 2.7 Study selection and screening

We downloaded all titles and abstracts retrieved by electronic searching (Web of Science, Pubmed, Google Scholar) to the reference management software. Duplicates were removed using the Rayyan tool, and three authors (IO, AO and OA) independently screened the titles and abstracts of the articles.

We obtained the full text of potentially relevant studies. The eligible articles were retrieved and independently screened by three (3) authors (IO, AO and OA). Cohen’s Kappa screening (Excel sheet designed to screen the selected articles) was used to evaluate the inter-rater agreement between two data extractors by two authors (IO & AO) to ensure the data was void of bias and accurate. At each phase of the screening, it was ensured that there was an agreement between the three authors on the selected articles that will be included in the study, and cases of conflict were resolved by a third author (AO). We strictly followed the article selection process to answer the research questions, and to achieve the study objectives.

### 2.8 Data extraction

Microsoft Excel spreadsheet was used for data extraction. An Excel spreadsheet was created to organize and store the extracted data. A column was set up for each data point or variable for extraction. These include the reference, citation, journal name, title of the journal, first author, year, country, study setting, type of study, age, sex, species, number of specimens used, therapeutic effects of *Balanites aegyptiaca*, part of the plant used, bioactive phytochemicals, mechanism of action, dosage, primary outcome, test statistics used, study limitations/future work. Data were extracted from the full text of the included articles using the Proforma (the excel data extraction sheet) and entered into the corresponding columns. The extracted data were cross-checked to ensure consistency and accuracy.

### 2.9 Data cleaning

Data cleaning was done to delete any errors or duplications. We used Excel features like sorting, filtering, and grouping to manage and analyze the extracted data. We also used multiple sheets within the spreadsheet to separate data extraction, analysis, and results. Five authors (GA, IO, OO, AO and OA) participated in screening the selected full-text articles and cross-checking the extracted data.

### 2.10 Quality assessment

The quality of the papers included in the study were assessed by two authors (AO, OA) using the Joanna Briggs Institute Case Control Checklist. The checklist assesses the methodological quality of case-control studies based on ten questions (S3 File). Possible responses were ‘yes’, ‘no’, or ‘maybe’. We assigned a maximum score of 1 to each question, with a potential minimum score of 0 and a maximum score of 10 for each article. However, before the quality assessment, we decided not to exclude any study based on the quality rating only.

## 3 Results

### 3.1 Selection of studies

**Figure 1:**
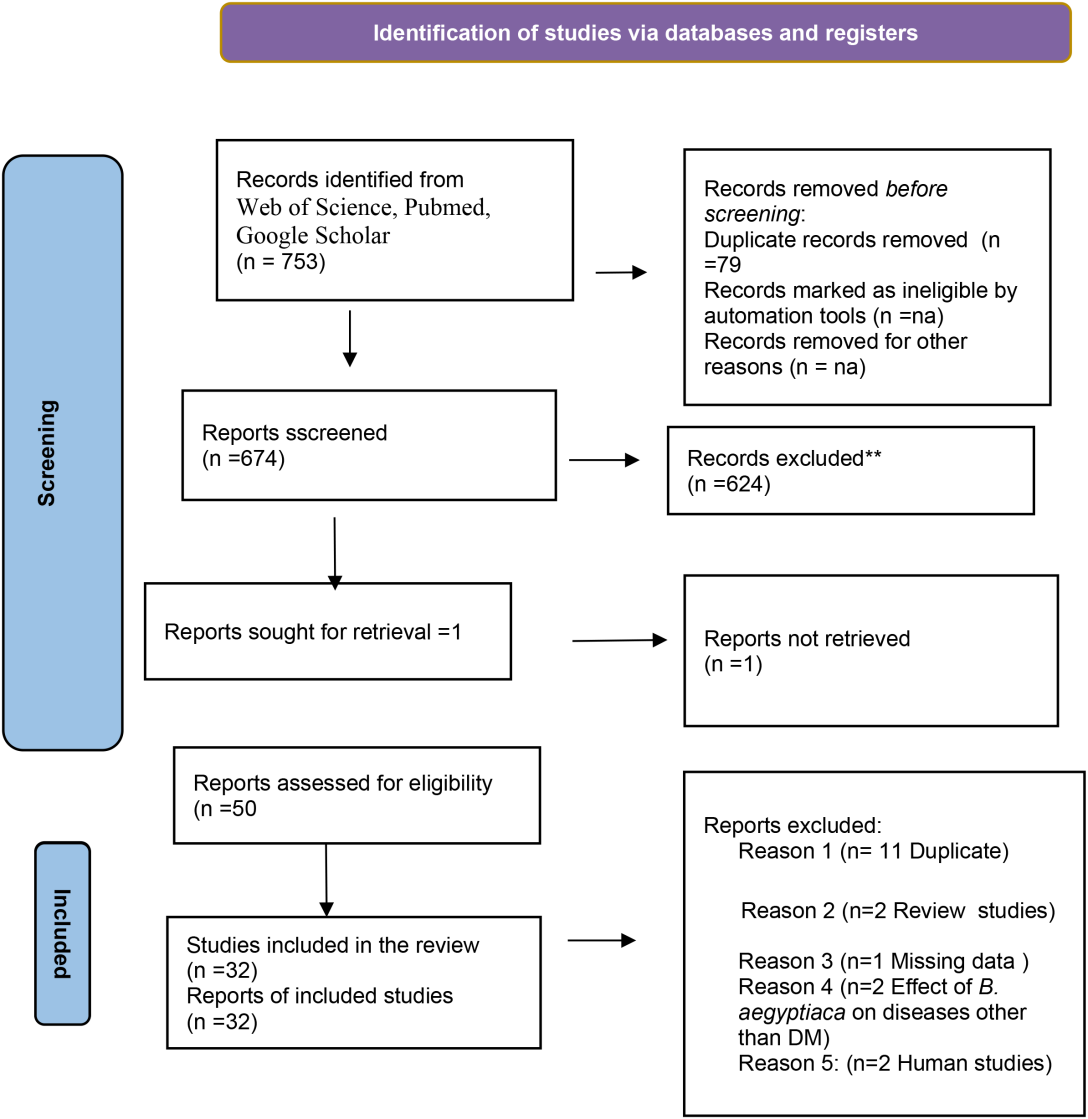
PRISMA flow diagram of the process of study identification and selection.

We identified 753 articles through three databases (Web of Science, PubMed and Google Scholar). After removing seventy nine (79) duplicates studies, the remaining 674 articles were screened using their abstracts and titles and 624 articles were excluded. The full text manuscript of the remaining 50 articles were obtained for full text evaluation. Eighteen of the full text manuscript were further excluded because they were either duplicates, review studies, non-retrievable manuscript, Human studies or were researches with outcome different from the study objectives. Only thirty two (32) articles were found eligible and were included in the study (**Figure 1**).

The PRISMA flow chart for our study is presented in **Figure 1**.

### 3.2 Narrative synthesis

All the 32 studies in this review were animal experiment, mostly involved Streptozotocin-Induced diabetic Rats and non-diabetic induced controls. The included studies were conducted across various countries in Africa and the Middle East. Eighteen studies (56.3%) from Egypt (16–29, 42–45), one (3.1%) from Ethiopia (46), eight studies (25%) from Nigeria (31–35, 40, 47, 48) and five studies (15.6%) from Saudi Arabia (30, 36–39).

In over 20 years, from 1986 to 2015, only six (18.8%) of these experimental studies on rats were conducted (16, 20, 22, 24, 38, 49). A significant rise in research activity occurred between 2016 and August 2024, during which 26 studies were published. Various parts of the *Balanites aegyptiaca* tree were utilized in these studies, including leaves, bark, fruits, seeds, and kernels. However, the fruit mesocarp was the most frequently used, accounting for 35% of the studies.

The predominant extraction method was aqueous extraction, although other solvents such as hydroethanol, hydromethanol, and non-polar solvents (e.g., ethyl acetate, chloroform, and hexane) were also used. Numerous phytochemicals were identified in the plant, and a few studies advanced further to isolate and characterize the active compounds responsible for the plant’s hypoglycemic effects.

In these animal studies, the dosage used to mitigate hyperglycaemia ranges from 10 - 1,500 mg/kg body weight. These were presented as capsules, raw fruit mesocarp, or infusions. For most of the studies, hypoglycemia was achieved from the 14th day of treatment, while a few observed a decrease in hyperglycaemia a few hours after the first dosage was administered. The summary of the characteristics of the included studies is presented in **Table 2**.

**Table 2:** Characteristics of the included studies.

SN	Reference	Title of Article	Country	Type of Study	Part of the plant used	Phytochemicals Present	Solvent used for extraction	Dosage form/dose given
1.	Abdel-Mageed et al., 2019 (23)	Evaluation of Antidiabetic Potentiality of Truffles and Balanites Aegyptiaca among Streptozotocin-Induced Diabetic Rats	Egypt	Animal Experiment	Plant materials (parts not specific)	Not mentioned	95% Ethanol	200mg/kg BW
2.	Helal et al., 2013 (24)	Antidiabetic and Antihyperlipidemic Effect of Balanites aegyptiacaSeeds (Aqueous Extract) on Diabetic Rats	Egypt	Animal Experiment	Seed	Not mentioned	Aqueous	0.42mg/kg BW
3.	Ghanem et al., 2016 (25)	The effect of herbal tea from Balanites aegyptiaca fruits on streptozotocin-induced diabetes mellitus in rats	Egypt	Animal Experiment	Fruit	Not mentioned	Aqueous	0.25 - 1.0%
4.	Ibrahim et al., 2024 (26)	Balanites aegyptiaca dates hydroethanolic extract shows anti-neurodegenerative effect in diabetes-mediated neurodegenerative disorders in rats	Egypt	Animal Experiment	Fruit (Mesocarp)	Not mentioned	70% ethanol	2250mg/kg
5.	Zaakouk et al., 2018 (27)	Effect Of Balanites Aegyptiaca (Heglig Dates) And Persea Americana (Avocado Fruit) On Some Hematological And Biochemical Parameters In Streptozotocin-Induced Diabetic Male Rats	Egypt	Animal Experiment	Not indicated	Not mentioned	Not mentioned	100 mg/kg BW
6.	Abou Khalil et al., 2016 (29)	Antidiabetic and Antioxidant Impacts of Desert Date (Balanites aegyptiaca) and Parsley (Petroselinum sativum) Aqueous Extracts: Lessons from Experimental Rats	Egypt	Animal Experiment	Fruits pericarp	Not mentioned	Aqueous	1,500 mg/kg BW daily
7.	Ismail et al 2022 (17)	Balanites aegyptiaca (Heglig Dates) Reduces Oxidative Stress, and Biophysical Alterations of Erythrocyte Membranes in Streptozotocin-Induced Diabetic Rats	Egypt	Animal Experiment	Fruits flesh	Not mentioned	Aqueous	100 mg/kg
8.	Gad 2006 (49)	Biochemical study of the antidiabetic action of the Egyptian plants Fenugreek and Balanites	Egypt	Animal Experiment	Fruits mesocarp	Diosgenin	Aqueous	1,500mg/kg
9.	Mhya et al., 2018 (34)	Antihyperglycemic Effect of Balanites aegyptiaca Leaves Extract-Fractions in Stretozotocin-Induced Diabetic Rats	Nigeria	Animal Experiment	Leaves	Not mentioned	Aqueous Fraction	400 mg/kg
10.	Mhya et al., 2016 (33)	Evaluation of Hypoglycemic Potential of Extracts of Balanites Aegyptiaca Parts	Nigeria	Animal Experiment	Leaves, stem bark and fruit mesocarp	Phenols and flavonoids	Aqueous Fraction	400 mg/kg
11.	Watad et al., 2023 (18)	Protective effects of Balanites aegyptiaca extract, MSCs and Exosome against Diabetic nephropathy in male albino rats	Egypt	Animal Experiment	Fruits mesocarp	Not mentioned	Aqueous	80 mg/kg
12.	Mhya et al., 2016 (32)	Effects of Aqueous Fraction of Ethanolic Extract of Balanites aegyptiaca stem bark on glucose metabolic enzymes in Streptozotocin-induced Diabetic Rats	Nigeria	Animal Experiment	Stem bark	Phenols and flavonoids	Aqueous Fraction	400 mg/kg
13.	Mhya et al., 2018 (31)	Effect of Extract-Fractions of Balanites aegyptiaca Fruit-Mesocarp on Glucose metabolizing Enzymes in Diabetic Rats	Nigeria	Animal Experiment	Fruit mesocarp	Not mentioned	Aqueous Fraction	400 mg/kg
14.	Al-Thobaiti et al., 2019 (30)	Hepatoprotective and antioxidant effects of methanolic extracts of Balanites aegyptiaca against streptozotocin-induced liver damage in rats	Saudi Arabia	Animal Experiment	Kernel and mesocarp	Not mentioned	Methanol	650 mg/kg
15.	Gamde et al., 2023 (40)	Histologic and Biochemical Effect of Balanite aegyptiaca Fruit Extract on Alloxan-induced Diabetes in Wistar rats	Nigeria	Animal Experiment	Fruits pericarp	Not mentioned	Aqueous	100–300 mg/kg
16.	Farid et al., 2024 (19)	Desert date seed extract‐loaded chitosan nanoparticles ameliorate hyperglycemia and insulin deficiency through the reduction in oxidative stress and inflammation	Egypt	Animal Experiment	Seed	Not mentioned	80% ethanol	10–20 mg/kg
17.	Nadro, MS et al., 2014 (48)	The effects of Balanite aegyptiaca kernel cake as supplement on alloxan-induced diabetes mellitus in rats	Nigeria	Animal Experiment	Kernel	Not mentioned	Not applicable	Not applicable
18.	Ogori et al., 2022 (35)	The effect of Balanities aeqyptiaca defatted protein meal and protein concentrate supplemented diet on biochemical and molecular stability of diabetic wister albino rat	Nigeria	Animal Experiment	Kernel	Flavonoids, saponins, cavacrol, cinnary alcohols	Not applicable	Not applicable
19.	Ogori et al., 2022 (47)	Enzymatic protein hydrolysates from aduwa (Balanites aeqyptiaca L) seed meal supplemented diet on α-amylase, α-glucosidase and antioxidants activity of Streptozotocin-induced diabetic wister albino rat	Nigeria	Animal Experiment	Seed	Not mentioned	Not applicable	Not applicable
20.	Alakilli, 2016 (37)	Fruit extract nanoparticles increase the efficiency of Balanites sp against diabetes mellitus in albino male rats	Saudi Arabia	Animal Experiment	Fruit	Not mentioned	Aqueous	20-42mg/kg BW
21.	Bussa et al., 2017 (46)	The Potential of Camel Milk and Extracts of Major Plants Browsed by the Animal for Diabetes Treatment	Ethiopia	Animal Experiment	Not mentioned	Not mentioned	90% Methanol	Not mentioned
22.	Barakat et al 2023 (21)	The potential role of exosome-derived mesenchymal stem cells and Balanites aegyptiaca in diabetic nephropathy amelioration in rats	Egypt	Animal Experiment	Fruit mesocarp	Not mentioned	Aqueous	80mg/kg body weight
23.	Kamel et al., 1991 (22)	Studies on Balanites aegyptiaca Fruits,An antidiabetic Egyptian folk medicine	Egypt	Animal Experiment	Fruit mesocarp	Steroidal glycosides, flavonol glycosides, steroidal saponins	Aqueous	Not mentioned
24.	El-Saadany et al 1986 (20)	Biochemical Action of Balanites aegyptiaca Fruits as a Possible Hypoglycemic Agent	Egypt	Animal Experiment	Fruit mesocarp	Saponins	80% ethanol	Not mentioned
25.	Al-Malki 2015 (38)	Management of Hyperglycemia by Ehylacetate extract of Balanites aegyptiaca (desert date)	Saudi Arabia	Animal Experiment	Fruit	Syringic acid, vanilic acid beta sitosterol	Ethylacetate	10-50mg/kg BW
26.	Ezzat et al., 2017 (28)	*In-vitro* & *In-vivo* antidiabetic potential of extracts & furostanol saponin from Balanites aegyptiaca	Egypt	Animal Experiment	Fruit pericarp	Furostanol saponin	Chloroform fraction, ethyl butanol fraction, aqueuous fraction	100 -200mg/kg BTW
27.	Al-Thabaiti and Zeid 2019 (39)	Antidiabetic potential of Balanites aegyptiaca kernel flesh and their combination against Streptozotocin induced hyperglcemia in male rats	Saudi Arabia	Animal Experiment	Fruit mesocarp and Kernel	Not mentioned	Methanol	650mg/kg
28.	El Deib and Ali 2018 (36)	Molecular investigation of Antidiabetic effect of Balanites aegyptiaca fruit on streptozotocin-induced diabetic rats	Saudi Arabia	Animal Experiment	Fruit mesocarp	Not mentioned	Aqueous	Not mentioned
29.	Hassanin et al., 2018 (42)	*Balanites aegyptiaca* ameliorates insulin secretion & decreases pancreatic apoptosis in diabetic rats;Role of SAPK/JNK pathway	Egypt	Animal Experiment	Fruit mesocarp	Pure saponins, Balanittin 1,3; diosgenin, stigmast-4-en-3-ol, balanitin-2;	70% ethanol cold	50mg/kg BW
30.	Zaky et al 2022 (45)	Antidiabetic effects & modes of action of the *Balanites aegyptiaca* fruit and seed aqueous extract in NA/STZ-induced diabetic rats	Egypt	Animal Experiment	Fruits & Seeds	3,4,6-tri-O-methyl-d-glucose; Triethylphosphine	Aqueous	200mg/kg BW
31.	Baragob et al., 2013 (16)	The hypoglycemic effect of aqueous extract of fruit of *Balanites aegyptiaca* in Alloxan -induced diabetic rats	Egypt	Animal Experiment	Fruits	Rutin, interketones organic compounds, oils (volatile and fatty acids),saponins	Ethanol and Aqueous	200-800mg/kg BW
32.	Mosaad et al. (44) 220	BAAE-AgNPs Improve Symptoms of Diabetes in STZ-induced Diabetic Rats	Egypt	Animal Experiment	Seed	Not mentioned	Aqueous	600-2,000mg/kg BW

### 3.3 Study outcome

The summary of the study outcome is presented in **Table 3**. Treatment with *B. aegyptiaca* showed a significant decrease in blood glucose levels of the experimental rats induced with streptozotocin (STZ) or alloxan-induced diabetes. Aside from regulating blood glucose, other outcomes observed include improved lipid profile, increased body weight, and insulin production. These all buttressed the antidiabetic effects of *B. aegyptiaca*. Some studies also demonstrated that treatment with *B. aegyptiaca* ameliorated most of the toxic effects of Alloxan and STZ used to induce diabetes in experimental rats (42, 49, 50). Some studies showed that *B. aegyptiaca* fruit mitigated hyperglycaemia via inhibition of oxidative stress demonstrated by inhibition of lipid peroxidation in diabetic rats (17, 19). Three of the studies further revealed that *B. aegyptiaca* not only had a hypoglycemic effect in diabetic rats but also improved liver and kidney functions, which are frequently impaired in diabetes (24, 30, 40).

**Table 3:** Outcomes of the included studies.

S/no	Extractor	Type of study	Proposed mechanism of action	Therapeutic effect of *Balanites aegyptiaca*	Duration of efficacy of BA
1.	Abdel-Mageed et al.	Experimental	Truffle and *Balanites aegyptiaca* extract caused a significant decrease in IL-1β and iNOS genes mRNA expression, which can combat the cytotoxic effect of STZ on pancreatic β-cells reflected in lower FGB levels, and the restoration of pancreatic histopathological parameters of diabetic rats that received the extract compared to the positive control diabetic group.	Treatment with Truffle and *B. aegyptiaca* significantly decreased oFBG level and restorative effect on both islets ‘cells and acinar cells than that achieved by *B. aegyptiaca* alone.	15 days
2.	Helal et al	Experimental	Treatment with water extract of *B. aegyptiaca* seeds ameliorated most β-cells dysfunction and increased the insulin’s receptors sensitivity that is associated by improvement in general diabetic conditions.	Reduction in hyperglycemia, increased insulin level, improve lipid profile, and increased liver glycogen content	30 days
3.	Ghanem et al	Experimental	Treatment with *B. aegyptiaca* tea; blood glucose, urea, creatinine, AST, ALT, total cholesterol and triglyceride was significantly reduced in diabetic rats.	Reduction in body weight; mitigation of hyperglycemia, hypoinsulinemia, dislipidemia,	4 weeks
4.	Ibrahim et al	Experimental	*B. aegyptiaca* showed a significant reduction in blood glucose and serum glucagon concentrations, a significant increase in serum insulin, and remarked melioration in the serum lipid profile. Biochemical, histopathological, and immunohistochemical analyses demonstrated these results.	Reduction in body weight, mitigation of hyperglycemia, hypoinsulinemia, dislipidemia, anti neurodegenerative effects	45 days
5.	Zaakouk et al	Experimental	The results showed a significant decrease in total protein and albumin levels in diabetic controlgroup when compared to the treated group. It is also revealed insignificant changes in all treated groups in comparison to the diabetic control group.	Reduction in body weight, mitigation of hyperglycemia, hypoinsulinemia, dislipidemia, anti neurodegenerative effects	30 days
6.	Abou- Khalil	Experimental	The herbal preparations significantly reduced the mean plasma glucose and MDA levels and significantly increased the mean plasma insulin, L-PK, and TAC levels in the treated diabetic groups compared to the diabetic control group.	Reduction in body weight, mitigation of hyperglycemia, hypoinsulinemia, dislipidemia, anti neurodegenerative effects	45 days
7.	Ismail et al	Experimental	In the diabetes group, there was a significant increase in the auto-oxidation rate of haemoglobin (hemolysis) compared to control, while the auto-oxidation rate was decreased significantly in the rats treated orally with Balanites aegyptiaca whe	Hypoglycemic and antioxidative effects	30 days
8.	Gad et al	Experimental	*B. aegyptiaca* extract reduced blood glucose level by 24% and significantly decreased liver glucose-6-phosphatase activity in diabetic rats.	Hypoglycemic and antioxidative effects	21 days
9.	Mhya et al.	Experimental	Treating diabetic rats with extract-fractions of *B. aegyptiaca* leaves slightly elevated serum insulin, lowered fasting blood glucose levels and improved serum lipid profile; total cholesterol, TG, LDL-C and VLDL-C toward normal.	Reduce fasting blood glucose, increased insulin level, improved lipid profile	28 days
10	Mhya et al.	Experimental	*In vivo* hypoglycemic effects of solvent fractions of Balanites aegyptiaca were observed. The plant solvent-fractions caused a reduction in blood glucose levels in a time-dependent manner. The hypoglycemic activity of the plant was further substantiated by its improved glucose tolerance in the normalglycemic/diabetic treated rats. It suggested that the plant solvent fractions contain compounds that can correct impaired glucose tolerance in diabetes, exhibiting antidiabetic effects.	Increased glucose tolerance	Not stated
11.	Watad et al	Experimental	Reduced blood sugar, increased insulin level, and improved lipid profile	Reduced blood sugar, increased insulin level, and improved lipid profile	4 weeks
12.	Mhya et al.	Experimental	A non-significant reduction in blood sugar	Reduced blood sugar, improved lipid profile and regulated glucose metabolic enzymes	28 days
13.	Mhya et al.	Experimental	Treatment with the ethanol extract-fractions of Balanites aegyptiaca parts lowered fasting blood glucose levels, reversed serum total cholesterol, triglyceride, low-density lipoprotein cholesterol, very low-density lipoprotein cholesterol, high-density lipoprotein levels, serum albumin, total protein and ix regulate glucose metabolic enzymes activities.	Regulated glucose metabolic enzymes	28 days
14.	Al-Thobaiti and Zeid	Experimental	Reduced blood sugar and improved liver function	Reduced blood sugar and improved liver function	6 weeks
15.	Gamde et al	Experimental	Reduced blood sugar, improved liver and kidney functions	Reduced blood sugar, improved liver and kidney functions	2 ways (14 weeks)
16.	Farid et al	Experimental	Reduced blood glucose, increased insulin, reduced pancreatic oxidative stress and inflammation	Reduced blood glucose, increased insulin, reduced pancreatic oxidative stress and inflammation	8 weeks
17.	Nadro. and Samson.	Experimental	Reduced blood glucose levels	Antihyperglycaemic and antihyperlipidemic effect	3 weeks
18.	Ogori et al	Experimental	The meals from defatted and protein concentrate inhibit α-amylase and α-glycosidase inhibitory activity *in vivo*.	Reduce blood glucose; Reduce levels of lipids; increase insulin production	
19.	Friday	Experimental		Blood glucose profile showed improvements with BA treatment.	
20.	Alakilli	Experimental		Oral administration of *B. aegyptiaca* significantly decreased the blood glucose, alterations in the expression of insulin and gluconeogenic genes, DNA Fragmentation	
21.	Bussa et al	Experimental			
22.	Barakat et. al	Experimental & RCT	*B. aegyptiaca* extract has a hypoglycemic action mediated by insulin-mimetic action. Enhanced sensitivity for insulin receptors, potentiation and stimulation of insulin secretion, inhibition of intestinal glucosidase activity, acceleration of glucose metabolism, suppression of hepatic gluconeogenesis and improved hepatic glycogen storage	Hypoglycemic activity, increased insulin activity, glucose metabolism and improved hepatic glycogen storage	72 Hours
23.	Kamel et al	Experimental	Reduced blood glucose level and increases hexokinase activity	Reduced blood glucose level and increases hexokinase activity	4 Hours
24.	El-Saadany et al	Experimental	Increased body weight, decreased blood glucose and improved liver function	Increased body weight, decreased blood glucose and improved liver function	10 days
25.	Al-Malki et al	Experimental	Decreased levels of inflammation makers,	Reduce blood glucose; Reduce levels of lipids; reduce weight; increase insulin production.	
26.	Ezzat et al	Experimental	Increased biosynthesis of insulin;	Reduce blood glucose; Reduce levels of lipids; reduce weight; increase insulin production	2 weeks
27.	Al-Thabaiti et al	Experimental	Enhanced pancreatic secretion of insulin from the β-cells; Glucose homeostasis	Reduce blood glucose; Reduce levels of lipids; reduce weight; increase insulin production	6 weeks
28.	El Deib and Ali	Experimental with approved ethics	Induction of glucose transport GLUT-2 & GLUT -4; Increases pyruvate kinase (LPK) responsible for liver glucose utilization; catalyzes the conversion of phospoenolpyruvate; Reduction in mRNA expression of pancreatic α-amylase	Reduce blood glucose; Reduce levels of lipids; reduce weight; increase insulin production	4 weeks
29.	Hassanini et al	Experimental	BA extracts inhibited oxidative stress & inhibition of SAPK-JNK pathway; Decreased apoptosis in of pancreatic β-cells, reduced fasting plasma glucose and glycated haemoglobin	Reduce blood glucose; Reduced levels of lipids; reduce weight; increased insulin production	1 month
30.	Zaky.,et al	Experimental	Reduced HDL cholesterol level; increased insulin sensitivity; increased number of β-cells; demonstrates hypoglycemic activity, hypolipidemia, insulinotropic activity associated with a reduction in oxidative stress; enhancement of antioxidant defence system, reduced apoptosis in pancreatic β-cells; improvement in insulin secretion response	Reduce blood glucose; Improve lipids profile; reduce weight; increase insulin secretion	4 weeks
31.	Baragob., et al	Experimental	Hypoglycemic effect	Reduce blood glucose; Improve lipids profile; reduce weight; increased insulin secretion	
32.	Mosaad et al	Experimental	Down-regulated pancreatic TGF-β1 and Akt gene expression in diabetic rats and resulted in the regulation of hepatic GLUT-2, as well as an increase in the regulation of hepatic GK and pancreatic B-Cl2 gene expression.	Reduced plasma glucose, improved lipid profile, improved body weight, insulin homeostas,glycated haemoglobin level, antioxidant activity and reduced inflammation.	12 weeks

Three studies (36,37,39) also reported neurodegenerative effect when diabetes induced rats were treated with the *B. aegyptiaca extra.*

### 3.4 SRYCLE risk of bias assessment –

We use the Systematic Review Centre for laboratory animal experiment (SRYCLE) risk of bias assessment for the risk of bias assessment in this study.

The SYRCLE Risk of Bias (RoB) is a comprehensive framework used in the assessment of methodological quality of animal studies (51). The assessment for this study has ten domains which assessed either selection, performance, detection, attrition or a reporting bias (**Table 4**).

**Table 4:** SRYCLE Risk of bias for the assessment.

S/no	Domain	Assessment question
1	sequence generation	Is the allocation sequence adequately generated and applied?
2	Baseline characteristics	Were the rats similar at baseline or were they adjusted for confounders in the analysis
3	Allocation concealment (selection bias)	Was the allocation adequately concealed?
4	Random housing	Were the rats randomly housed during the experiment?
5	Blinding (Performance bias)	Were the investigators blinded from knowledge which intervention each animal received during the experiment?
6	Random outcome assessment	Were animals selected at random for outcome assessment?
7	Detection bias: Blinding outcome assessors	Was the outcome assessor blinded?
8	Attrition bias: Incomplete outcome data	Were incomplete outcome data adequately addressed?
9	Reporting bias: Selective outcome reporting	Are reports of the study free of selective outcome reporting?
10	Other source of bias	Was the study apparently free of other problems that could result in high risk of bias? (*)

The SYRCLE RoB would adjudge low-risk for a particular domain if the answer to the domain question was “Yes”. A ‘high score’ would be allocated if the answer was “NO” The scoring would be ‘unclear risk’ if there were no sufficient information to make judgement (51).

The assessment revealed that in twenty-nine (90.6%) of the study, the allocation sequence was adequately generated and applied. Baseline Characteristics of the experimental rats were similar in all (100%) cases. Allocation concealment was reported in only 2 (6.25%) studies (38, 53). Only twenty-one (65.6%) studies reported random housing.

**Figure 2:**
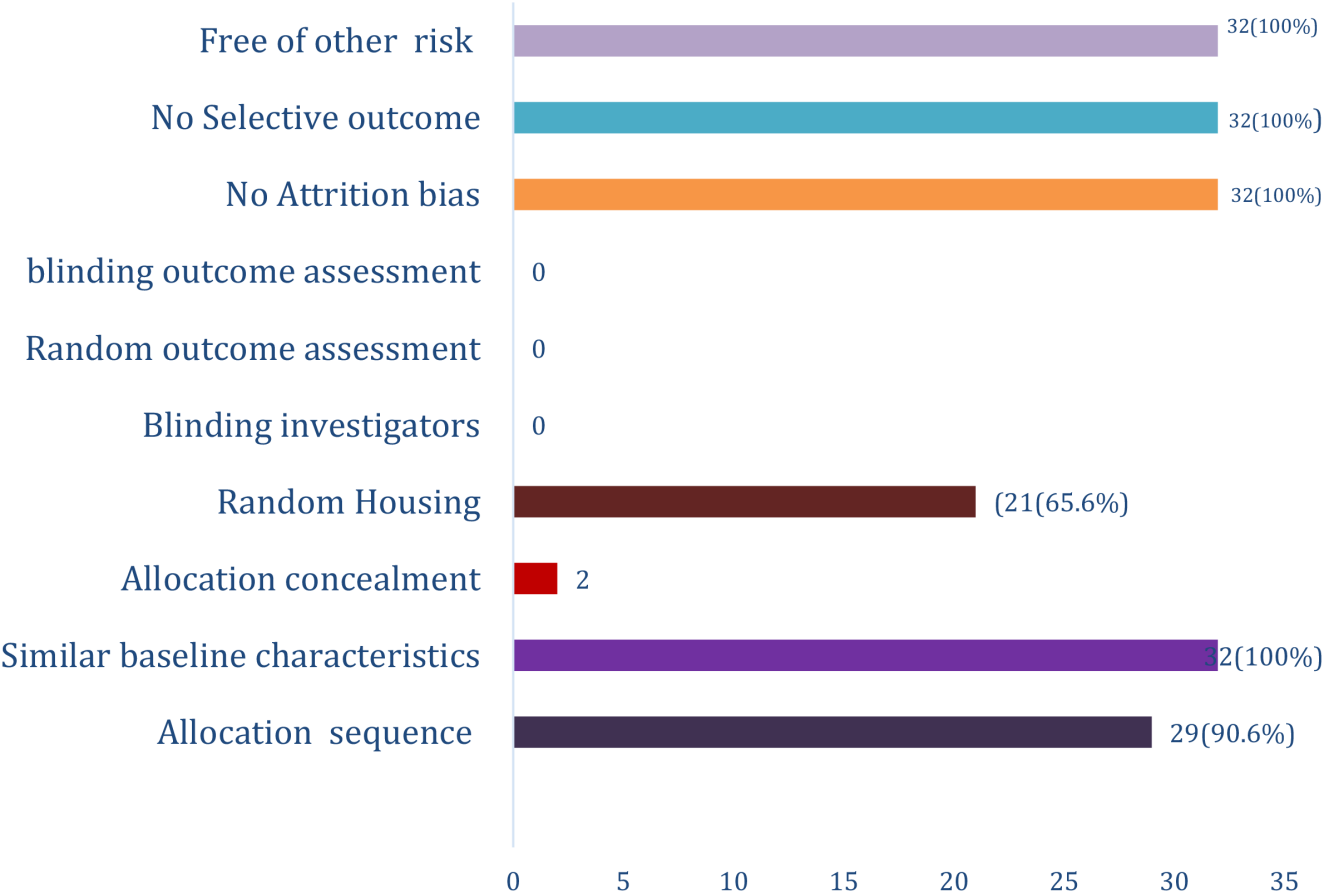
SYRCLE risk of bias assessment for Therapeutic Effects of *Balanites aegyptiaca* DEL Extract on Diabetes Mellitus: A Systematic Review.

No study reported blinding of the investigators and random outcome assessment and blinding of the outcome assessors. There was no attrition bias, no selective outcome reporting. All the studies were apparently free of other problems that could result in high risk of bias (**Figure 2**).

## 4 Discussion

*Balanites aegyptiaca*, a plant widely distributed in Africa, is used in traditional African medicine to manage various diseases, including diabetes. The use of *B. aegyptiaca* has recently attracted significant attention from scientists, leading to an increase in the number of studies investigating its antidiabetic effects in the last test years. This systematic review shows that the use of the Balanites aegyptiaca plants for animals induced diabetes produced antidiabetic effects in all the animal studies. This preclinical study therefore showed the potential of *B. aegyptiaca* to mitigate diabetes mellitus in human subjects.

Over the ages, human beings have relied on plants as a source of therapeutic agents, and this has attracted scientific attention. Hence, much effort has been directed toward studying the medicinal effects of these plants and investigating the underlying mechanisms and bioactive phytochemicals responsible for the observed medicinal. Our findings shows that a significant hypoglycemic effect was recorded in both Alloxan and STZ-induced diabetes. Other antidiabetic-related effects observed with *B. aegyptiaca* include improved lipid profile (24–29, 32–35, 39, 45), increased insulin production (16, 19, 21, 22, 24, 38, 39, 42, 44), changes in serum protein and increased glycogen content (21). Also of interest is that virtually all parts of the plants- the seed kernel, fruit mesocarp, leaf, and stem barks from the tree showed a hypoglycemic effect. However, a study comparing the activity of stem bark, leaf, and fruit mesocarp reported higher activity for the leaf and fruit mesocarp than the stem (33).

The medicinal effects of plants have been attributed to their phytochemical constituents, which can modulate different targets and pathways (52, 53–55). Various phytochemicals were identified in this plant using different spectrometry methods, which have identified various phytochemicals, showing that the plant is rich in phytochemicals (50). Specific flavonoids and saponin compounds were isolated from the plant. The flavonoids were inactive, while the saponins showed antidiabetic effects. The saponin compounds namely; 26-O-beta-D-glucopyranosyl-(25R)-furost-5-ene-377beta,22,26-triol 3-O-[alpha-L-rhamnopyranosyl-(1—2)]-[beta-D-xylopyranosyl-(1—3)]-[alpha-L-rhamnopyranosyl-(1—4)]-beta-D-glucopyranoside, 26-O-beta-D-glucopyranosyl-(25R)-furost-5-ene-beta,22,26-triol 3-O-(2,4-di-O-alpha-L-rhamnopyranosyl)-beta-D-glucopyranoside, 26-(O-β-d-glucopyranosyl)-22-O-methylfurost-5-ene-3β,26-diol-3-O-β-d-glucopyranosyl-(1 → 4)-[α-l-rhamnopyranosyl-(1 → 2)]-β-d-glucopyranoside individually exhibited lower antidiabetic effect compared to the active fraction (22, 42). However, when the saponin compounds were combined, the combined saponin showed a greater antidiabetic effect (22). This implies that the pooled saponin in the plant is responsible for its antidiabetic effect as the individual saponins either work synergistically or additively to produce the antidiabetic effect.

Studies have also investigated the mechanisms underlying the antidiabetic effects of *B aegyptiaca*. Reported mechanisms include antioxidant (17, 21, 22, 25, 29, 35, 38, 44, 47, 50), anti-inflammatory (22, 23, 38, 44), regulation of glucose/carbohydrate metabolism (32, 33, 49, 56–58), mitigation of insulin resistance (50), inhibition of pancreatic α-amylase (35, 36, 47) and inhibition intestinal α-glycosidase (35, 47), modulation of glucose transporter (36, 44) and apoptosis (44).

We observed in this study that there was low risk of bias for allocation sequence, similarity of the animals at baseline, attrition bias, random housing, selective outcome and other risk of bias There were however High risk of bias noted for blinding and random outcome assessment and blinding of the investigators. This risk of bias assessment tool gave a fair critical appraisal of evidence from this animal studies (45, 59).

### 4.1 Strengths of the study

This systematic review utilized an extensive search strategy of the database sources.

The inclusion and exclusion criteria were also clearly pre- defined. The PRISMA flow diagram reveals the rigorous process for the article selection, it shows that the methodology were reproducible, ensures standardization, transparency and clarity of the data generation process. The SYRCLE risk of bias specifically applies to animal studies, helps to improve study validity and the research quality.

### 4.2 Limitation of the study

Geographical spread of the study was limited; they were skewed to few countries in Africa and Asia.

## 5 Conclusion

The study found that *B. agyptiaca* had antidiabetic effects in the experimental animals. All parts of the plants showed antidiabetic effect, but the leaves and fruit showed superior activity. The plant worked by modulating different targets and pathways. Therefore, clinical studies on the antidiabetic effect of *B. aegyptiaca in humans* are recommended to determine the antidiabetic effects and the usefulness of the *B. agyptiaca* as a therapeutic agent in human with diabetes mellitus.

## Data Availability

The original contributions presented in the study are included in the article/**Supplementary Material**. Further inquiries can be directed to the corresponding author.
