# Supplementary material for: Therapeutic effects of Balanites aegyptiaca DEL extract on diabetes mellitus: a systematic review
Source: Front Clin Diabetes Healthc. 2025 Sep 2;6:1651789. doi: 10.3389/fcdhc.2025.1651789 (PMC12436142; doi:10.3389/fcdhc.2025.1651789)
Supplement: Supplementary file 1 [file SupplementaryFile1.pdf]

The PRISMA flow chart for our study is presented in Fig 2

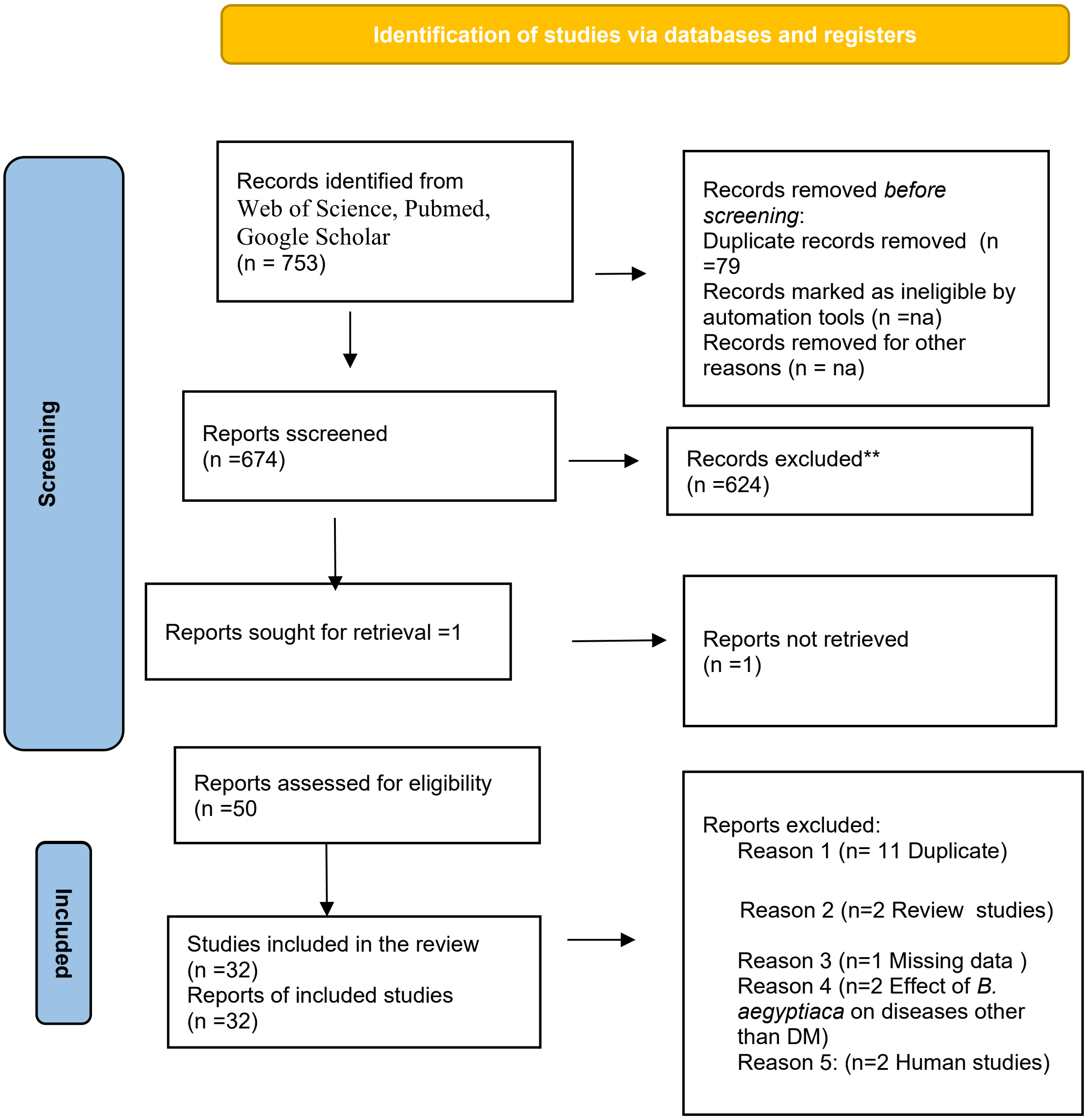

Fig 2. PRISMA flow diagram of the process of study identification and selection
