# Supplementary material for: Therapeutic effects of Balanites aegyptiaca DEL extract on diabetes mellitus: a systematic review
Source: Front Clin Diabetes Healthc. 2025 Sep 2;6:1651789. doi: 10.3389/fcdhc.2025.1651789 (PMC12436142; doi:10.3389/fcdhc.2025.1651789)
Supplement: Supplementary file 2 [file SupplementaryFile2.pdf]

PubMed Search strategy

For the PubMed search, we used medical subject headings (MeSH) keywords and free text, which were combined with the Boolean operators “AND” and “Or.”
Keywords related to “therapeutic effect”, “*Balanites aegyptiaca*” and “diabetes mellitus”, were used. Diabet*Search term include “Therapeutic effect” OR “Medicinal effect” OR “Benefits” OR “Biological activity” OR “Side effect” OR “Therapeutic biological activity” OR “Antidiabetic” AND “Balanites aegyptiaca” OR “Desert date” AND “Diabetes mellitus” OR “Type 2 diabetes” OR “Type 1 diabetes” OR “Non-insulin-dependent diabetes” OR “Diabet*”. Only studies in cell lines and animal models were included.
Publications in all languages were included and google translator was used for interpretation. Our search included every language. The therapeutic effect was defined as hypoglycemic effect and/or increase in insulin activity.
