# Supplementary material for: Therapeutic effects of Balanites aegyptiaca DEL extract on diabetes mellitus: a systematic review
Source: Front Clin Diabetes Healthc. 2025 Sep 2;6:1651789. doi: 10.3389/fcdhc.2025.1651789 (PMC12436142; doi:10.3389/fcdhc.2025.1651789)
Supplement: Supplementary file 3 [file Table2.docx]

**Supplementary 3.**

**Quality Assessment of the Included Studies**

| **SN** | Title  Title of Journal | Were the groups comparable other than the presence of disease in cases or the absence of disease in controls? | Were cases and controls matched appropriately? | Were the same criteria used for identification of cases and controls? | Was exposure measured in a standard, valid and reliable way? | Was exposure measured in the same way for cases and controls? | Were confounding factors identified? | Were strategies to deal with confounding factors stated? | Were outcomes assessed in a standard, valid and reliable way for cases and controls? | Was the exposure period of interest long enough to be meaningful? | Was appropriate statistical analysis used? | Total  Score | Level of Bias |
| --- | --- | --- | --- | --- | --- | --- | --- | --- | --- | --- | --- | --- | --- |
|  | Evaluation of Antidiabetic Potentiality of Truffles and *Balanites aegyptiaca* among Streptozotocin Induced Diabetic Rats | 1 | 1 | 1 | 1 | 1 | 1 | 1 | 1 | 1 | 1 | 10 | Low |
|  | Antidiabetic and Antihyperlipidemic Effect of Balanites aegyptiaca Seeds (Aqueous Extract) on Diabetic Rats | 1 | 1 | 1 | 1 | 1 | 1 | 1 | 1 | 1 | 1 | 10 | Low |
|  | The effect of herbal tea from Balanites aegyptiaca fruits on streptozotocin-induced diabetes mellitus in rats | 1 | 1 | 1 | 1 | 1 | 1 | 1 | 1 | 1 | 1 | 10 | Low |
|  | Balanites aegyptiaca dates hydroethanolic extract shows anti-neurodegenerative effect in diabetes-mediated neurodegenerative disorders in rats | 1 | 1 | 1 | 1 | 1 | 1 | 1 | 1 | 1 | 1 | 10 | Low |
|  | Effect Of Balanites aegyptiaca (Heglig Dates) And Persea Americana (Avocado Fruit) On Some Hematological And Biochemical Parameters In Streptozotocin Induced Diabetic Male Rats | 1 | 1 | 1 | 1 | 1 | 1 | 1 | 1 | 1 | 1 | 10 | Low |
|  | Antidiabetic and Antioxidant Impacts of Desert Date (Balanites aegyptiaca) and Parsley (Petroselinum sativum) Aqueous Extracts: Lessons from Experimental Rats | 1 | 1 | 1 | 1 | 1 | 1 | 1 | 1 | 1 | 1 | 10 | Low |
|  | Balanites aegyptiaca (Heglig Dates) Reduces Oxidative Stress, and Biophysical Alterations of Erythrocyte Membranes in Streptozotocin-Induced Diabetic Rats | 1 | 1 | 1 | 1 | 1 | 1 | 1 | 1 | 1 | 1 | 10 | Low |
|  | Biochemical study of the antidiabetic action of the Egyptian plants Fenugreek and Balanite | 1 | 1 | 1 | 1 | 1 | 1 | 1 | 1 | 1 | 1 | 10 | Low |
|  | Antihyperglycemic Effect of Balanites aegyptiaca Leaves Extract-Fractions in Stretozotocin-Induced Diabetic Rats | 1 | 1 | 1 | 1 | 1 | 1 | 1 | 1 | 1 | 1 | 10 | Low |
|  | Evaluation of Hypoglycemic Potential of Extracts of Balanites Aegyptiaca Parts | 1 | 1 | 1 | 1 | 1 | 1 | 1 | 1 | 1 | 1 | 10 | Low |
|  | Protective effects of Balanites aegyptiaca extract, MSCs and Exosome against Diabetic nephropathy in male albino rats | 1 | 1 | 1 | 1 | 1 | 1 | 1 | 1 | 1 | 1 | 10 | Low |
|  | Effects of Aqueous Fraction of Ethanolic Extract of Balanites aegyptiaca stem bark on glucose metabolic enzymes in Streptozotocin-induced Diabetic Rats | 1 | 1 | 1 | 1 | 1 | 1 | 1 | 1 | 1 | 1 | 10 | Low |
|  | Effect of Extract-Fractions of Balanites aegyptiaca Fruit-Mesocarp on Glucose metabolizing Enzymes in Diabetic Rats | 1 | 1 | 1 | 1 | 1 | 1 | 1 | 1 | 1 | 1 | 10 | Low |
|  | Hepatoprotective and antioxidant effects of methanolic extracts of Balanites aegyptiaca against streptozotocin-induced liver damage in rats | 1 | 1 | 1 | 1 | 1 | 1 | 1 | 1 | 1 | 1 | 10 | Low |
|  | Histologic and Biochemical Effect of Balanite aegyptiaca Fruit Extract on Alloxan_induced Diabetes in Wistar rats | 1 | 1 | 1 | 1 | 1 | 1 | 1 | 1 | 1 | 1 | 10 | Low |
|  | Desert date seed extract‐loaded chitosan nanoparticles ameliorate hyperglycemia and insulin deficiency through the reduction in oxidative stress and inflammation | 1 | 1 | 1 | 1 | 1 | 1 | 1 | 1 | 1 | 1 | 10 | Low |
|  | The effects of Balanite aegyptiaca kernel cake as supplement on alloxan-induced diabetes mellitus in rats | 1 | 1 | 1 | 1 | 1 | 1 | 1 | 1 | 1 | 1 | 10 | Low |
|  | The effect of Balanities aeqyptiaca defatted protein meal and protein concentrate supplemented diet on biochemical and molecular stability of diabetic wister albino rat | 1 | 1 | 1 | 1 | 1 | 1 | 1 | 1 | 1 | 1 | 10 | Low |
|  | Enzymatic protein hydrolysates from aduwa (Balanities Aeqyptiaca L) seed meal supplemented diet on α-amylase, α-glucosidase and antioxidants activity of Streptozotocin-induced diabetic wister albino rat | 1 | 1 | 1 | 1 | 1 | 1 | 1 | 1 | 1 | 1 | 10 | Low |
|  | Fruit extract nanoparticles increase the efficiency of Balanites sp against diabetes mellitus in albino male rats | 1 | 1 | 1 | 1 | 1 | 1 | 1 | 1 | 1 | 1 | 10 | Low |
|  | The Potential of Camel Milk and Extracts of Major Plants Browsed by the Animal for Diabetes Treatment | 1 | 1 | 1 | 1 | 1 | 1 | 1 | 1 | 1 | 1 | 10 | Low |
|  | The potential role of exosome-derived mesenchymal stem cells and Balanites aegyptiaca in diabetic nephropathy amelioration in rats | 1 | 1 | 1 | 1 | 1 | 1 | 1 | 1 | 1 | 1 | 10 | Low |
|  | Studies on Balanites aegyptiaca Fruits,An antidiabetic Egyptian folk medicine | 1 | 1 | 1 | 1 | 1 | 1 | 1 | 1 | 1 | 1 | 10 | Low |
|  | Biochemical Action of Balanites aegyptiaca Fruits as a Possible Hypoglycemic Agent | 1 | 1 | 1 | 1 | 1 | 1 | 1 | 1 | 1 | 1 | 10 | Low |
|  | Management of Hyperglycemia by Ehylacetate extract of Balanites aegyptiaca (desert date) | 1 | 1 | 1 | 1 | 1 | 1 | 1 | 1 | 1 | 1 | 10 | Low |
|  | In-vitro & In-vivo antidiabetic potential of extracts & furostanol saponin from Balanites aegyptiaca | 1 | 1 | 1 | 1 | 1 | 1 | 1 | 1 | 1 | 1 | 10 | Low |
|  | Antidiabetic potential of Balanites aegyptiaca kernel flesh and their combination against Streptozotocin induced hyperglcemia in male rats | 1 | 1 | 1 | 1 | 1 | 1 | 1 | 1 | 1 | 1 | 10 | Low |
|  | Molecular investigation of antidiabetic effect of Balanites aegyptiaca fruit on streptozotocin-induced diabetic rats | 1 | 1 | 1 | 1 | 1 | 1 | 1 | 1 | 1 | 1 | 10 | Low |
|  | *Balanites aegyptiaca* ameliorates insulin secretion & decreases pancreatic apoptosis in diabetic rats;Role of SAPK/JNK pathway | 1 | 1 | 1 | 1 | 1 | 1 | 1 | 1 | 1 | 1 | 10 | Low |
|  | Antidiabetic effects & modes of action of the Balanites aegyptiaca fruit and seed aqueous extract in NA/STZ-induced diabetic rats | 1 | 1 | 1 | 1 | 1 | 1 | 1 | 1 | 1 | 1 | 10 | Low |
|  | The hypoglycemic effect of aqueous extract of fruit of Balanites aegyptiaca in Alloxan -induced diabetic rats | 1 | 1 | 1 | 1 | 1 | 1 | 1 | 1 | 1 | 1 | 10 | Low |
|  | BAAE-AgNPs Improve Symptoms of Diabetes in STZ-induced Diabetic Rats | 1 | 1 | 1 | 1 | 1 | 1 | 1 | 1 | 1 | 1 | 10 | Low |
